# Fifteen years of progress in understanding frailty and health in aging

**DOI:** 10.1186/s12916-018-1223-3

**Published:** 2018-11-27

**Authors:** Kenneth Rockwood, Susan E. Howlett

**Affiliations:** 1 Geriatric Medicine, Department of Medicine, Dalhousie University, Halifax, NS Canada; 2 Department of Pharmacology, Dalhousie University, Halifax, NS Canada

**Keywords:** Frailty index, Frailty phenotype, Deficit index, Deficit accumulation, Aging, biomarkers, Senescence, senolytics

## Abstract

The notion of frailty has evolved for more than 15 years. Although there is no consensus definition, frailty reflects a state of increased vulnerability to adverse health outcomes for individuals of the same chronological age. Two commonly used clinical tools, the frailty index and the frailty phenotype, both measure health-related deficits. The frailty index is a ratio of the number of deficits that an individual has accumulated divided by all deficits measured, whereas the phenotype specifies frailty as represented by poor performance in three of five criteria (i.e., weight loss, exhaustion, weakness, slowness, lack of activity). From human studies, animal models of both approaches have been developed and are beginning to shed light on mechanisms underlying frailty, the influence of frailty on disease expression, and new interventions to attenuate frailty. Currently, back-translation to humans is occurring. As we start to understand subcellular mechanisms involved in damage and repair as well as their response to treatment, we will begin to understand the molecular basis of aging and, thus, of frailty.

## Background

A 1994 paper summarized frailty as “*an evolving concept*” [1] and, to some extent, this remains true. Herein, we summarize progress in research on frailty over the last 15 years. We consider one of the first uses of the term and contrast it with the two main approaches now used as determined by an extensive literature search on iterative uses of frailty, focusing on definitions, animal models, prevention, and treatment. Classic papers or reviews were prioritized where useful.

### Our still evolving understanding of frailty

Research regarding frailty saw the beginning of a new era approximately 15 years ago. Prior to 2001, a PubMed search for the word ‘frailty’, when it was defined at all, was defined by its statistical meaning. To statisticians, frailty had been generalized to quantify variability in the risk of adverse outcomes for people with the same degree of exposure, and thus its origin was rooted in demography [2]. Unmeasured heterogeneity of aging rates was invoked to explain the apparent paradox that, at extreme old age, the risk of death fell. The reasoning was that, prior to the eleventh decade, mortality risk was averaged across people with varying intrinsic rates of aging; however, past this age, only very slow agers remained alive, and thus the mortality rate appeared to slow. In 2001, two distinct clinical approaches to understanding frailty were elaborated [3, 4]. One of these approaches argues that the increased risk of frailty could be captured as a characteristic syndrome, understood as the loss of strength, speed, or weight, a sense of lack of energy, or the inability to perform demanding activities such as gardening and heavy housework. Combinations of at least three of these features are held to define a ‘frailty phenotype’ [3]. Conversely, the other clinical approach (developed by our team) defines frailty not as a specific syndrome, but rather as a state of age-related deficit accumulation, quantified in a ‘frailty index’, itself representing the ratio of deficits present to deficits evaluated for a given individual [4]. To the consternation of those who argue that progress requires a majority consensus, both operational definitions still stand. (Our view is that progress requires an approach that can resolve the contradictions between the two).

Nevertheless, despite some contradictions – chiefly regarding which individuals are classified as frail, and in their abilities to stage frailty and predict mortality – the two approaches to frailty have much in common [5]. Each identifies people at increased risk, albeit often not the same people. They have both been operationalized for use in administrative databases [6, 7], with one being used routinely in primary care for that purpose [8]. Additionally, both approaches are widely used [9], including in special populations such as people living with HIV/AIDS [10], as well as in multi-omics analyses of frailty [11, 12] or to define risk in invasive or toxic healthcare interventions [13, 14]. Finally, they have both been used to evaluate the association of frailty with the subsequent onset of other well-defined, late-onset illnesses, including dementia [15]. Both approaches are also used with substantial modification [16, 17].

In short, the operationalization of frailty, even with two competing approaches, has allowed a wide range of studies to be undertaken in older adults at increased risk. Similarly, the ability to quantify frailty in people is a major development that has led to recent advances in our understanding of the biology of frailty.

### Biological models and mechanisms of frailty

Whether measured as a phenotype or by deficit accumulation, frailty has demonstrable biological underpinnings that can be studied with animal models [18]. Both the frailty index and frailty phenotype approaches have been adapted for use in naturally aging animals. The first mouse frailty index was developed in 2012, where scores that deviated from average values in healthy adults for blood work, activity levels, body composition, and hemodynamics were counted as deficits [19]. This was followed by observational ‘clinical’ frailty index tools developed for use in both mice and rats [20, 21]. The frailty phenotype approach has also been tailored for use in aging rodents [22]. Together, these powerful new tools have the potential to help translate advances in basic science into meaningful clinical interventions.

There is strong evidence that aging mice can model frailty in humans, at least with respect to the frailty index. Properties of the frailty index (e.g., ranges of frailty scores, rates of deficit accumulation, and submaximal limits to frailty) are similar in mice and people across the life course [23]. In addition, naturally aging female mice have higher frailty scores than males, as seen in clinical studies [24]. Mouse models have also inspired new approaches to frailty assessment in people. For instance a frailty index based on routine blood work and hemodynamic measures (the FI-LAB), which was originally developed for use in mice [19], has been shown to predict mortality in people [25]. This approach has also provided a sound basis for understanding that frailty can be seen across the life course, with determinants that arise prior to clinically detectable deficits [26, 27]. Other translational work has studied epigenetic changes – characterized as the DNA methylation clock – both in frailty as a deficit accumulation [28] and in the frailty phenotype [29].

Advances in understanding subcellular mechanisms of damage and repair are providing new knowledge on the molecular basis of aging and, thus, of frailty. Animal models are providing insights on the physiologic mechanisms of frailty, the influence of frailty on disease, and interventions to mitigate frailty. For example, pro-inflammatory cytokine levels are positively correlated with frailty in aging mice in a sex-specific fashion, indicating a critical role for inflammation in frailty [24]. Signs of cardiac aging, including myocardial hypertrophy and contractile dysfunction, are also graded by the frailty index score [30], suggesting that heterogeneity in overall health can help explain heterogeneity in cardiac aging. The mouse frailty index also declines in response to interventions designed to extend lifespan (e.g., resveratrol treatment, caloric restriction) and health-span (e.g., angiotensin converting enzyme inhibitors) of mice [31, 32].

Various hallmarks of aging appear to be susceptible to interventions. One such intervention involves a new therapeutic class of senolytic drugs, which selectively induce apoptosis in senescent cells and thereby reduce secretion of deleterious senescence-associated cytokines and related damaging agents. A recent report suggested that intermittent oral administration of a ‘senolytic cocktail’ can selectively eliminate senescent cells, and thereby decrease their secretion of frailty-related proinflammatory cytokines [33]. Drugs that can counter the senescence-associated secretory phenotype (known as SASP) include metformin, which is now in human trials. Further, signaling between the mitochondrial and nuclear genomes to offer adaptive responses during aging appear to be highly conserved, offering additional treatment targets [34].

Nevertheless, despite the shared multiplicity of uses, it has been expressed that the two approaches to frailty have distinct purposes, or contexts, in which they are most likely to be feasible, and should therefore be employed in that regard [35]. Another view is that real progress requires treatment and that regulatory approval of treatment requires a clear operational definition, arguing that the possibility of regulatory approval would be a trumping virtue. Conversely, it is sobering to note that, in a review of frailty in acute care [9], the most common operational definition was none at all.

A major sign of the growing importance attached to frailty and its widespread use is the recent pushback that has been observed – many older people do not like the term [36]. Therefore, the use of alternative terms have been proposed, such as ‘resilience’, yet this has also proved a controversial definition [37]. Our view is that we should not set aside the progress made in a formal quantitative understanding of frailty in order to engage in a debate about semantics. Indeed, there are formal approaches to resilience (in engineering disciplines), each with a considerable mathematical apparatus that can be employed [37]; adapting insights from quantitative disciplines has been done in relation to frailty [27].

Equally concerning is the occasional sense that frailty screening is being promoted as a means to root out the undeserving elderly ill – screening for frailty to prevent admission of those who might otherwise ‘block’ hospital beds rather than to improve care by, for example, making routine care less hazardous. The goal of defining frailty is not simply to operationalize risk. Instead, for frailty assessment to help patients, information from it must inform the care plan. This has been seen, for example, in orthogeriatrics, where the dual geriatric assessment goals of evaluation and management have proven to benefit both lifespan and health-span [38]. Finally, both views of frailty have strong social determinants and consequences [39]; their interaction itself faces controversy in how the social determinants of health are operationalized. Progress in this regard, as with frailty itself, can be demonstrated despite the elusiveness of a final consensus on how to define the social determinants of health.

## Conclusions

The concept of frailty aims to address the considerable heterogeneity in health status of people as they age. Two operational approaches are in routine use and await overarching research to explain the ways in which they differ. Nevertheless, they have much in common, both identifying older adults at greater risk.

Now that links of frailty to aging mechanisms are becoming available, a better method to assay heterogeneity in risk may emerge, possibly even accompanied by new therapeutic approaches that tackle the fundamental processes of aging. However that may pan out, variable impacts of aging that give rise to frailty will continue to involve a strong set of social determinants. The important challenge posed by older people with multiple and interacting medical and social problems will remain to be addressed, regardless of the words used to describe them. Therefore, all knowledge on frailty will be essential.
